# Supplementary material for: eHealth adoption and use among healthcare professionals in a tertiary hospital in Sub-Saharan Africa: a Qmethodology study
Source: PeerJ. 2019 Apr 19;7:e6326. doi: 10.7717/peerj.6326 (PMC6476286; doi:10.7717/peerj.6326)
Supplement: Appendix S1 [file peerj-07-6326-s001.docx]

**CRIB SHEET**

# FACTOR 1 ARRAY CRIB SHEET

**Factor 1 has seven significantly loading participants and explains 13% of the study variance. It has an eigenvalue of 4.68. Five of the loading participants are physicians and two are nurses. There are two females and five males with an average age of 37.7 years.**

# Item ranked at +6

#5: If the CIS is extended I would use it

# Items ranked higher in Factor 1 array than other arrays

| No | Statement | **Rank** |
| --- | --- | --- |
| 33 | Clinical information systems improves work efficiency | **+5** |
| 40 | My gender affects my use of the clinical information systems | **-5** |
| 4 | Superiors at work think I should use the clinical information systems | **-1** |
| 1 | It is easy to remember how to perform tasks with the clinical information systems | **+2** |
| 10 | Using clinical information systems makes caring for patients easier | **+4** |
| 46 | The clinical information systems is not compatible with other platforms I use | **-1** |
| 37 | People who influence my clinical behaviour think I should the system | **+2** |
| 24 | My age has nothing to do with my ability to use the clinical information systems effectively | **+5** |
| 43 | I hesitate to use the clinical information systems for fear of making mistakes I cannot correct | **0** |
| 35 | Patients/families like it when i uses the clinical information system | **+1** |
| 15 | Patients/families believe clinical information systems use reduces chances of medication errors | **+2** |

# Items ranked lower in Factor array 1 than any other

| No | Statement | **Rank** |
| --- | --- | --- |
| 26 | It is easy for me to become skilful at using clinical information systems | **+1** |
| 21 | The use of clinical information systems is pertinent to my various related tasks | **0** |
| 39 | Using clinical information systems enables me to accomplish tasks more quickly | **+3** |
| 31 | I could complete the job using the clinical information systems if there was no one around to tell me what to do as I go | **0** |
| 16 | It is easy to get the system to do what I want it to do | **-2** |
| 11 | Management support staff innovations on clinical information systems use in the workplace | **-5** |
| 25 | My use of clinical information systems is entirely under my control | **-3** |
| 36 | My ICT experience affects my use of the clinical information system | **-1** |
| 32 | There is availability of technical assistance for clinical information systems in my hospital | **-5** |
| 22 | The clinical information systems are clear and understandable | **-3** |
| 38 | There are available resources to use the clinical information system | **-4** |

# Item ranked at -6

#28: Management organise regular training on the use of clinical information systems at the work place

# FACTOR 2 CRIB SHEET

**Factor 2 has seven significantly loading participants and explains 13% of the study variance. It has an eigenvalue of 4.68. Five of the loading participants are physicians and two are nurses. There are two females and five males within this Factor and have an average age of 42.6 years.**

# Item ranked at +6

#9: Using clinical information systems facilitates better patient care decision making

# Items rank higher in Factor 2 array than other arrays

| No | Statement | **Rank** |
| --- | --- | --- |
| 40 | My gender affects my use of the clinical information systems | **-5** |
| 7 | Using clinical information systems improves my performance | **+5** |
| 6 | Using clinical information systems increases my productivity | **+4** |
| 2 | Using clinical information systems improves patient care | **+5** |
| 45 | My use of the clinical information system is specific to the task i want to carry out | **+2** |
| 28 | Management organise regular training on the use of clinical information systems at the work place | **-3** |
| 42 | Patients/families believe clinical information systems use is good for quality patient care | **+1** |
| 3 | Using clinical information systems reduces likelihood of medication error | **+2** |
| 36 | My ICT experience affects my use of the clinical information system | **+3** |
| 32 | There is availability of technical assistance for clinical information systems in my hospital | **-1** |
| 22 | The clinical information systems are clear and understandable | **+3** |
| 38 | There are available resources to use the clinical information system | **+1** |
| 19 | The senior management of this organization has been helpful in the use of the clinical information systems | **+2** |

# Items ranked lower in Factor array than other arrays

| No | Statement | **Rank** |
| --- | --- | --- |
| 33 | Clinical information systems improves work efficiency | **+4** |
| 4 | Superiors at work think I should use the clinical information systems | **-2** |
| 1 | It is easy to remember how to perform tasks with the clinical information systems | **0** |
| 31 | I could complete the job using the clinical information systems if there was no one around to tell me what to do as I go | **0** |
| 13 | The use of clinical information systems makes me apprehensive | **-5** |
| 17 | Interaction with the clinical information systems does not require a lot of mental effort | **-4** |
| 43 | I hesitate to use the clinical information systems for fear of making mistakes I cannot correct | **-5** |
| 8 | I am certain about the reliability of the information I get from the system | **-3** |
| 5 | If the clinical system is extended I would use it | **+1** |
| 23 | My use of clinical information systems is entirely voluntary | **0** |
| 30 | My routine tasks prevent me from having time to use the clinical information system | **-3** |

# Item ranked at -6

#14: Using the clinical information systems is a status symbol in my organization

# Factor 3 crib sheet

**Factor 3 has six significantly loading participants and explains 10% of the study variance. It has an eigenvalue of 3.6. Three of the participants are nurses and three are physicians. There are three females and three males within this Factor and they have an average age of 42.8 years.**

# Item ranked at +6

#23: My use of clinical information systems is entirely voluntary

# Items ranked higher in Factor 3 array than other arrays

| No | Statement | **Rank** |
| --- | --- | --- |
| 39 | Using clinical information systems enables me to accomplish tasks more quickly | **+5** |
| 31 | I could complete the job using the clinical information systems if there was no one around to tell me what to do as I go | **+3** |
| 6 | Using clinical information systems increases my productivity | **+4** |
| 13 | The use of clinical information systems makes me apprehensive | **-1** |
| 34 | Using clinical information systems is easier than other computer systems I use | **+2** |
| 41 | People who are important to me think I should use the clinical information systems | **+1** |
| 11 | Management support staff innovations on clinical information systems use in the workplace | **-1** |
| 28 | Management organise regular training on the use of clinical information systems at the work place | **-3** |
| 25 | My use of clinical information systems is entirely under my control | **+2** |
| 32 | There is availability of technical assistance for clinical information systems in my hospital | **-1** |
| 8 | I am certain about the reliability of the information I get from the system | **+3** |
| 14 | Using the clinical information systems is a status symbol in my organization | **0** |
| 30 | My routine tasks prevent me from having time to use the clinical information system | **+5** |

# Items ranked lower in Factor 3 array than other arrays

| No | Statement | **Rank** |
| --- | --- | --- |
| 33 | Clinical information systems improves work efficiency | **+4** |
| 4 | Superiors at work think I should use the clinical information systems | **-2** |
| 21 | The use of clinical information systems is pertinent to my various related tasks | **0** |
| 1 | It is easy to remember how to perform tasks with the clinical information systems | **0** |
| 10 | Using clinical information systems makes caring for patients easier | **+1** |
| 20 | Using clinical information systems increases my chance of getting a praise or reward | **-4** |
| 7 | Using clinical information systems improves my performance | **+2** |
| 2 | Using clinical information systems improves patient care | **+1** |
| 45 | My use of the clinical information system is specific to the task i want to carry out | **-2** |
| 42 | Patients/families believe clinical information systems use is good for quality patient care | **-3** |
| 3 | Using clinical information systems reduces likelihood of medication error | **-2** |
| 44 | The information in the system is always updated | **-4** |
| 12 | People in my organization who use the clinical information systems have more prestige than those who do not | **-5** |
| 35 | Patients/families like it when i uses the clinical information system | **-5** |
| 18 | Not having the clinical information system in some departments hinders my work in these areas | **-4** |
| 15 | Patients/families believe clinical information systems use reduces chances of medication errors | **-5** |

# Item ranked at -6

#40: My gender affects my use of the clinical information systems

# FACTOR 4 CRIB SHEET

**Factor 4 has eight significantly loading participants and explains 15% of the study variance. It has an eigenvalue of 5.4. Five of the participants are nurses and three are physicians. There are four females and four males within this Factor and they have an average age of 44.9 years.**

# Item ranked at +6

#29: Clinical Information systems are useful in the hospital

# Item ranked higher at Factor 4 array than other arrays

| No | Statements | **Rank** |
| --- | --- | --- |
| 33 | Clinical information systems improves work efficiency | **+5** |
| 26 | It is easy for me to become skilful at using clinical information systems | **+4** |
| 40 | My gender affects my use of the clinical information systems | **-5** |
| 21 | The use of clinical information systems is pertinent to my various related tasks | **+2** |
| 1 | It is easy to remember how to perform tasks with the clinical information systems | **+2** |
| 39 | Using clinical information systems enables me to accomplish tasks more quickly | **+5** |
| 20 | Using clinical information systems increases my chance of getting a praise or reward | **-1** |
| 16 | It is easy to get the system to do what I want it to do | **+3** |
| 27 | I always look for opportunities to use the system whenever I can | **+5** |
| 17 | Interaction with the clinical information systems does not require a lot of mental effort | **0** |
| 3 | Using clinical information systems reduces likelihood of medication error | **+2** |
| 44 | The information in the system is always updated | **+1** |
| 12 | People in my organization who use the clinical information systems have more prestige than those who do not | **0** |
| 18 | Not having the clinical information system in some departments hinders my work in these areas | **+4** |

# Items ranked lower at Factor 4 array than others

| No | Statements | **Rank** |
| --- | --- | --- |
| 4 | Superiors at work think I should use the clinical information systems | **-2** |
| 46 | The clinical information systems is not compatible with other platforms I use | **-4** |
| 6 | Using clinical information systems increases my productivity | **+1** |
| 34 | Using clinical information systems is easier than other computer systems I use | **-2** |
| 37 | People who influence my clinical behaviour think I should the system | **-2** |
| 41 | People who are important to me think I should use the clinical information systems | **-2** |
| 9 | Using clinical information systems facilitates better patient care decision making | **0** |
| 32 | There is availability of technical assistance for clinical information systems in my hospital | **-5** |
| 38 | There are available resources to use the clinical information system | **-4** |

# Item ranked at -6

#19: The senior management of this organization has been helpful in the use of the clinical information systems
