# Supplementary material for: eHealth adoption and use among healthcare professionals in a tertiary hospital in Sub-Saharan Africa: a Qmethodology study
Source: PeerJ. 2019 Apr 19;7:e6326. doi: 10.7717/peerj.6326 (PMC6476286; doi:10.7717/peerj.6326)
Supplement: Supplemental Information 2 — From QMN, Steven Brown, editor. [file peerj-07-6326-s003.pdf]

With all due respect to José Quiles and the authors of the Webler et al. *Primer*, there is no recommended minimum or maximum number of respondents in a Q study and no items-to-persons formula that can be said to apply. Abstract rules such as these almost invariably come from R factor analysis and from the logic of large numbers rather than from the logic of experimentation; they are then applied to Q simply because it is also factor-analytic, even though the reasoning for this may have been lost in translation.

To specify a preferred number of participants in a Q study is to grasp the stick from the wrong end; i.e., a determination of P-set size typically comes after other decisions are made. As a practical matter, a beginning is best made with a problem of some kind and with the kinds of persons who could be expected to have something relevant to say about the problem. In the case of Newman's (2005) study of doctor-assisted suicide, for instance, reliance was placed on Thompson's theory of public opinion, which holds that with respect to any controversy there will be some individuals who are experts, some who speak authoritatively (but without expert knowledge), some who represent special interests (i.e., who have a material stake in the outcome of the controversy), and some who speak as a function of their class background; and, of course, there are always the unknowledgeable. Also incorporating anticipated gender differences, this gives rise to the following factorial design:

A. INTERESTS (a) experts (b) authorities (c) special (d) class (e) unknowledgeable

B. GENDER (f) male (g) female

Experts would include individuals such as medical ethicists, thanatologists, sociologists and psychologists of death and dying, etc. Authorities would include priests, rabbis, and preachers, but also politicians and journalists, i.e., non-specialists who nevertheless render proclamations authoritatively due to their positions in society. Special interests might include physicians (who might also fall in the authority category), grief counselors, hospice workers, the terminally ill and their relatives and close friends. Class interests would include ordinary individuals from different social classes who lack any special interest in the outcome of the debate and who may be presumed to be responding from the value base that is characteristic of their class and social background. Unknowledgeables might include children or young adults who have yet to face the loss of even a pet.

These theoretical considerations give rise to 5x2 = 10 combinations, and here is where numbers now begin to play a role. Were we to select m=3 of each kind (i.e., 3 male experts, 3 male authorities, ..., and 3 female unknowledgeables), this would produce a P-set of size mAB = (3)(5)(2) = 30 individuals. The mathematics would be altered were we to introduce a third effect, e.g.:

C. AGE (h) young (i) middle (j) elderly

Now there are ABC = (5)(2)(3) = 30 combinations, of which we might elect to take 1 or 2 of each, for a P set of 30-60. We needn't be obsessive about complete balance. We might find it difficult, for instance, to find any elderly or even middle-aged individuals who might be considered unknowledgeable, which would result in empty cells in the P structure, but this is of little concern since we expect the functionality of the Q factors to supersede the structural characteristics of the P set. But the goal would be to make sure that no important group (e.g., authorities) is left unrepresented in the study.

The main purpose of the P-set structure is to provide diversity on the response side of the stimulus-response situation, comparable to the diversity provided by the the structure of the Q sample. Between

them, we expect these two structures, suitably replicated with items and persons, to reveal the main perspectives (i.e., Q factors) that are at issue. Hence, it is not numbers as such, but diversity that is the goal. There is, of course, the need to make sure where possible that the factors that emerge are suitably well defined, which usually means five or six pure cases with reasonably high factor loadings associated with each factor, but with any responses beyond the minimum being redundant. However, we rarely know in advance how many factors there will be nor which respondents will be defining each, hence the need to over-sample.

As to the items : persons ratio, I have never heard anyone make clear what the consequences might be for violating these ratios. Surely the world wouldn't come to an end. Neither the PQMethod nor the PCQ programs would explode if more cases than items were included. The factor analysis of the correlation matrix will still proceed (merrily so) without knowledge of the number of statements that produced the correlations. Moreover, the character of the raw scores in a Q-method study (which arise from interactions) is quite different from the character of scores in R (which are independent and non-interactive), and this alone undermines consideration of item : person ratios in Q methodology.

Reference

Newman, T.D. (2005). *Physician assisted suicide and euthanasia: A Q study of the links between ethics and public policy.* Doctoral dissertation, Kent State University.


```
_______________________________________________
*   ______   _______   _____   __  __   _____   ____  _    *   Steven R. Brown
|  |   ____||_     _||   _  ||   |  ||   _  ||    |  |    |   Political Science
|  |___   |   |   |   |  _|  |   |  ||  _|  |        |    |   Kent State University
|  |______|   |__|   |_____|  \___/  |_____||_|____|    |   (sbrown@kent.edu)
*___________________________________________________________* _____________________________
```


Time paradoxes will have given me a headache.

On 2/23/10 2:51 AM, "Karen Roberts" <karen.roberts-2@MANCHESTER.AC.UK> wrote:

Apologies for the very basic question but I'm new to Q and trying to
get my head around things.

Is there a recommended minimum number of participants required for a
Q-sort? I have read that around 40-60 participants is typical (Watts
& Stenner, 2005) but are there examples of studies using fewer
participants?

Should the number of participants at least equal the number of
statements in the Q-sort?

Any help would be most appreciated.
Kind regards,
Karen Roberts

--
Karen Roberts
Subject Leader PGCE Modern Languages
School of Education, Ellen Wilkinson Building (C2.20)
The University of Manchester

Oxford Road,Manchester,M13 9PL
Direct line/voicemail: 0161 275 3416
Fax: 0161 275 3528
PGCE Office: 0161 275 8472
Email: karen.roberts-2@manchester.ac.uk
